# Validation and comparison of the coding algorithms to identify people with migraine using Japanese claims data

**DOI:** 10.3389/fneur.2023.1231351

**Published:** 2023-11-27

**Authors:** Kentaro Yamato, Hiromi Sano, Koichi Hirata, Takeo Nakayama

**Affiliations:** ^1^Medical Affairs, Otsuka Pharmaceutical Co., Ltd., Osaka, Japan; ^2^Dokkyo Medical University, Tochigi, Japan; ^3^Department of Health Informatics, Graduate School of Medicine and School of Public Health, Kyoto University, Kyoto, Japan

**Keywords:** migraine, primary headache, predictive value, survey, claims data

## Abstract

**Purpose:**

The study aimed to validate and compare coding algorithms for identifying people with migraine within the Japanese claims database.

**Methods:**

This study used the administrative claim database provided by DeSC Healthcare, Inc., that was linked to the results of an online survey administered to adult users of the health app “kencom^®^.” The ability of the 12 algorithms to detect migraines using diagnostic records alone or with prescription records was evaluated based on sensitivity, specificity, positive predictive values (PPVs), and negative predictive values (NPVs). We used a migraine diagnosis judged based on respondents' self-reported symptoms according to the diagnostic criteria of the International Classification of Headache Disorders, version 3 (ICHD-3), as true.

**Results:**

Of the 21,480 individuals, 691 had migraine according to the ICHD-3 criteria. The 12 algorithms had a sensitivity of 5.4–8.8%, specificity of 98.8–99.6%, PPVs of 19.2–32.5%, and NPVs of 96.9–97.0%. Algorithm 9 (migraine diagnostic records more than once AND at least one prescription record for migraine prophylaxis or triptans in the same month as diagnosis) produced the highest PPV, whereas Algorithm 2 (at least one diagnostic record of migraine or tension-type headache) had the highest sensitivity. Similar trends were observed when using the ID-Migraine or 4-item migraine screener, instead of the ICHD-3 criteria, for case ascertainment.

**Conclusion:**

Strict algorithms, such as Algorithm 9, yielded a higher PPV but a lower sensitivity, and such algorithms may be suitable for studies estimating the relative risk. Conversely, algorithms based on a single diagnostic record, such as Algorithm 2, had a higher sensitivity and may be suitable for studies estimating the prevalence/incidence of disease. Our findings will help select a desirable algorithm for migraine studies using a Japanese claim database.

## 1 Introduction

Migraine is a highly disabling neurological disorder, with a prevalence exceeding that of diabetes, epilepsy, and asthma combined (1). It is more common in women than in men, with a global age-standardized prevalence of 18.9% in women and 9.8% in men (2). Estimates of the prevalence of migraine in Japan are slightly lower, ranging from 6.0 to 8.6% (3, 4). According to the Global Burden of Disease 2016 survey, it is the second leading cause of disability worldwide (1), particularly in women under the age of 50 years (2).

Despite its prevalence, migraines have only recently been recognized as an important public health concern (5). It was not included in the Global Burden of Diseases, Injuries, and Risk Factors studies before 2000 (2). Migraine research has historically been underfunded (6, 7) partially because it is an “invisible” disorder (8) and there are no obvious physical manifestations or clinical laboratory results associated with migraine. Consequently, this condition is often underestimated, underdiagnosed, and undertreated (9–12). However, its impact on productivity and quality of life is substantial (13). According to an employee population survey in Japan, the estimated annual economic loss due to presenteeism was USD 2,217 per person (5). Therefore, further research on migraine should be conducted in Japan to reduce this large patient burden.

The diagnosis of migraine is based on the symptoms reported by the patients. The diagnostic criteria for migraine, according to the International Classification of Headache Disorders, version 3 (ICHD-3), are attacks lasting for 4–72 h with any two of the following attributes: unilateral location, pulsating quality, moderate to severe pain intensity, and aggravation by physical activity (14). In addition, attacks must be accompanied by either nausea and/or vomiting or photophobia and phonophobia (14). Questionnaires such as the ID-Migraine (15) and 4-item migraine screener (16) have been developed and used as diagnostic screeners to aid in the diagnosis of migraine.

Medical claims databases, which generally store anonymized, individual-level, and standardized data on patients (e.g., age and sex) and claims (e.g., diagnosis, prescriptions, and treatments) in both inpatient and outpatient settings, are used in studies on many diseases in Japan; however, these data are not primarily generated for research purposes. Therefore, the use of validated algorithms to identify patients with a disease of interest is crucial to avoid misclassification of outcomes and exposures (17, 18), which can introduce bias (19). In validation studies, the accuracy of claims-based algorithms is usually compared to a gold-standard source of information such as clinical laboratory values, medical chart reviews, or registry data (20, 21). However, for migraine, which is diagnosed based on patients' descriptions of their symptoms, information obtained from a self-report questionnaire may help detect it. Indeed, a previous validation study conducted in the U.S. developed algorithms to identify patients who had not been diagnosed with chronic migraine using claims data. The study evaluated its performance using a questionnaire survey in combination with semi-structured interviews as the gold standard method for diagnosing chronic migraine (22).

In Japan, no studies have assessed claim-based algorithms for migraine detection. Therefore, in the present study, we compared 12 coding algorithms to identify people with migraines within a Japanese claim database based on four measures: sensitivity, specificity, positive predictive values (PPVs), and negative predictive values (NPVs). Given the diagnostic nature of migraine, which depends on patients' self-description of their symptoms, we used patient responses to an online survey asking about their symptoms, which incorporated questions in line with the diagnostic criteria of the ICHD-3. We considered migraine cases classified according to the survey responses as true migraine cases. Additionally, we have also examined the four measures considering individuals classified as having migraine according to the ID-Migraine and 4-item migraine screeners as true cases. Our findings could provide useful information to define and validate migraine cases in future database studies.

## 2 Materials and methods

This study used a combination of administrative claims data and the linked results of an online survey. The combined data were obtained from DeSC Healthcare, Inc. (DeSC). The details of the conduct of this study were provided in an earlier publication (23).

The claims data, covering the period from 1 December 2017 to 30 November 2020, were provided by the society-managed employment-based health insurance associations that had contracts with DeSC for subscribers who agreed to the secondary use of their medical data by DeSC (~600,000 subscribers). Data included patient information (e.g., age and sex), diagnoses, prescriptions, and treatments in both inpatient and outpatient settings.

Regarding the survey data, an online survey was administered by DeSC to the registered users of the health app “kencom^®^” aged 19–74 years (~150,000 users), irrespective of the presence of headache, from 1 to 30 November 2020. This health monitoring app was designed by DeSC and is freely available to users in Japan who are members of an affiliated society-managed, employment-based health insurance association (24). The survey questionnaire included items on sociodemographic characteristics (e.g., age, sex, residential area, occupation, and annual household income) and questions related to headache (the clinical features that include headache in the past 3 months, headache frequency, symptoms of headache, types of headache medicines used, impact of headache on daily activities, and questions to measure migraine-specific quality of life, work productivity, and activity impairment) (23). The questions also included items in line with the diagnostic criteria of the ICHD-3 (14) or common screening tools for migraine [e.g., ID-Migraine (15) and 4-item migraine screener (16)]. The study included all individuals who responded to the online survey regardless of whether they had headaches.

This study was approved by the independent ethics committee of Otsuka Pharmaceutical Co., Ltd. (approval no. 220617). The study used anonymized data, and no new individual-level consent was obtained for data use. The survey was conducted in accordance with the ethical guidelines for medical and biological research involving human subjects in Japan and the Declaration of Helsinki (revised in October 2013) of the World Medical Association.

### 2.1 Claims-based algorithms to identify people with migraine

The present study assessed the ability of 12 algorithms to detect people with migraines within the database (Table 1). These 12 algorithms were selected, through consultation with headache specialists and epidemiologists, from the combinations of the following disease and prescription codes identified in the claims data: diagnostic records of migraine [International Classification of Diseases-10 (ICD-10) code G43], tension-type headache (ICD-10 code G44.2), or premenstrual migraine (Japanese disease/injury code 8833260, under ICD-10 code N94.3) alone, or in combination with prescription records of acute medications [i.e., triptans, acetaminophen, and nonsteroidal anti-inflammatory drugs (NSAIDs)] or prophylactic medications (i.e., valproic acid, topiramate, propranolol, lomerizine, and candesartan). These medications were chosen from those listed in the Clinical Practice Guidelines for Headache 2021, according to the clinician's judgment on their use in migraine treatment in clinical practice (25). We constructed algorithms that included a diagnostic record of tension-type headache (G44.2), in addition to migraines, because chronic or frequent migraines can transform into tension-type headache (transformed migraines) and vice versa.

**Table 1:** Definitions of the 12 coding algorithms using claims data to detect people with migraine.

**Algorithm**	**Definition**
Algorithm 1	At least one (≥1) diagnostic record of migraine
Algorithm 2	At least one (≥1) diagnostic record of migraine or tension-type headache
Algorithm 3	Two or more (≥2) diagnostic records of migraine
Algorithm 4	The same diagnostic records of migraine or tension-type headache more than once (≥2)
Algorithm 5	At least one (≥1) prescription record for migraine prophylaxis (valproate, topiramate, propranolol, lomerizine, and candesartan) or triptans in the same month as the diagnostic record of migraine
Algorithm 6	At least one (≥1) prescription record for migraine prophylaxis (valproate, topiramate, propranolol, lomerizine, and candesartan) or triptans, acetaminophen, or NSAIDs in the same month as the diagnostic record of migraine
Algorithm 7	At least one (≥1) prescription record for migraine prophylaxis (valproate, topiramate, propranolol, lomerizine, and candesartan) or triptans in the same month as the diagnostic record of migraine or tension-type headache
Algorithm 8	At least one (≥1) prescription record for migraine prophylaxis (valproate, topiramate, propranolol, lomerizine, and candesartan) or triptans, acetaminophen, or NSAIDs in the same month as the diagnostic record of migraine or tension-type headache
Algorithm 9	Migraine diagnostic records more than once (≥2) AND algorithm 5
Algorithm 10	Migraine diagnostic records more than once (≥2) AND algorithm 6
Algorithm 11	The same diagnostic records of migraine or tension-type headache more than once (≥2) AND algorithm 7
Algorithm 12	The same diagnostic records of migraine or tension-type headache more than once (≥2) AND algorithm 8

NSAIDs, non-steroidal anti-inflammatory drugs.

### 2.2 Case ascertainment based on self-reported symptoms

As the database was not linked to patient medical records, we were unable to conduct a chart review, which is the gold standard for case ascertainment. Instead, the present study judged migraine cases based on respondents' self-reported symptoms according to the diagnostic criteria of the ICHD-3 (14). Additionally, we defined the cases according to two valid diagnostic screeners for migraine: (1) ID-Migraine, consisting of three items on disability, nausea, and photophobia (15); and (2) 4-item migraine screener, with four questions on the aggravation by daily activities, nausea/stomach discomfort, photophobia, and osmophobia (16). Conditions to meet the definitions of migraine according to each of these three criteria are provided in Supplementary Table S1.

### 2.3 Statistical analyses

The demographic and disease characteristics of the respondents are descriptively summarized. For each algorithm, a confusion matrix was constructed using a diagnosis based on survey responses as true (Table 2). The ability of each algorithm to detect people with migraine was evaluated primarily using PPV. The PPV was calculated as the proportion of migraine cases based on survey responses, i.e., true positives (= a), among migraine patients detected using the algorithm (= a + b) (Table 2). Additionally, sensitivity [proportion of true positives (a) among migraine cases based on survey responses (= a + c)], specificity [proportion of false positives (b) among non-migraine individuals based on survey responses (= b + d)], and NPV [proportion of true negatives (d) among non-migraine individuals based on the algorithm (= c + d)] were also calculated. For exploratory purposes, stratification by sex and age group (19–29, 30–39, 40–49, 50–59, and ≥60 years) was also performed. In addition to the analysis that meets the definition of migraine according to each of the three criteria, we have also performed analyses based on migraine cases that met all three criteria as well as those that met one of them. All statistical analyses were performed in SAS Release 9.4 (SAS Institute, Inc., NC, USA). Missing data were treated as they were, and no imputation was performed.

**Table 2:** Confusion matrix for calculation of assessment indices.

		**Meets the definition of migraine according to the ICHD-3 criteria, ID-Migraine, or 4-item migraine screener (“true” status of migraine)**	
		**Yes**	**No**
Meets the definition of migraine based on the claims-based algorithm	Yes	a	b
	No	c	d

a = frequency of “true positives,” b = frequency of “false positives,” c = frequency of “false negatives,” and d = frequency of “true negatives”. ICHD-3, International Classification of Headache Disorders, version 3.

## 3 Results

### 3.1 Disposition of respondents

**Figure 1:**
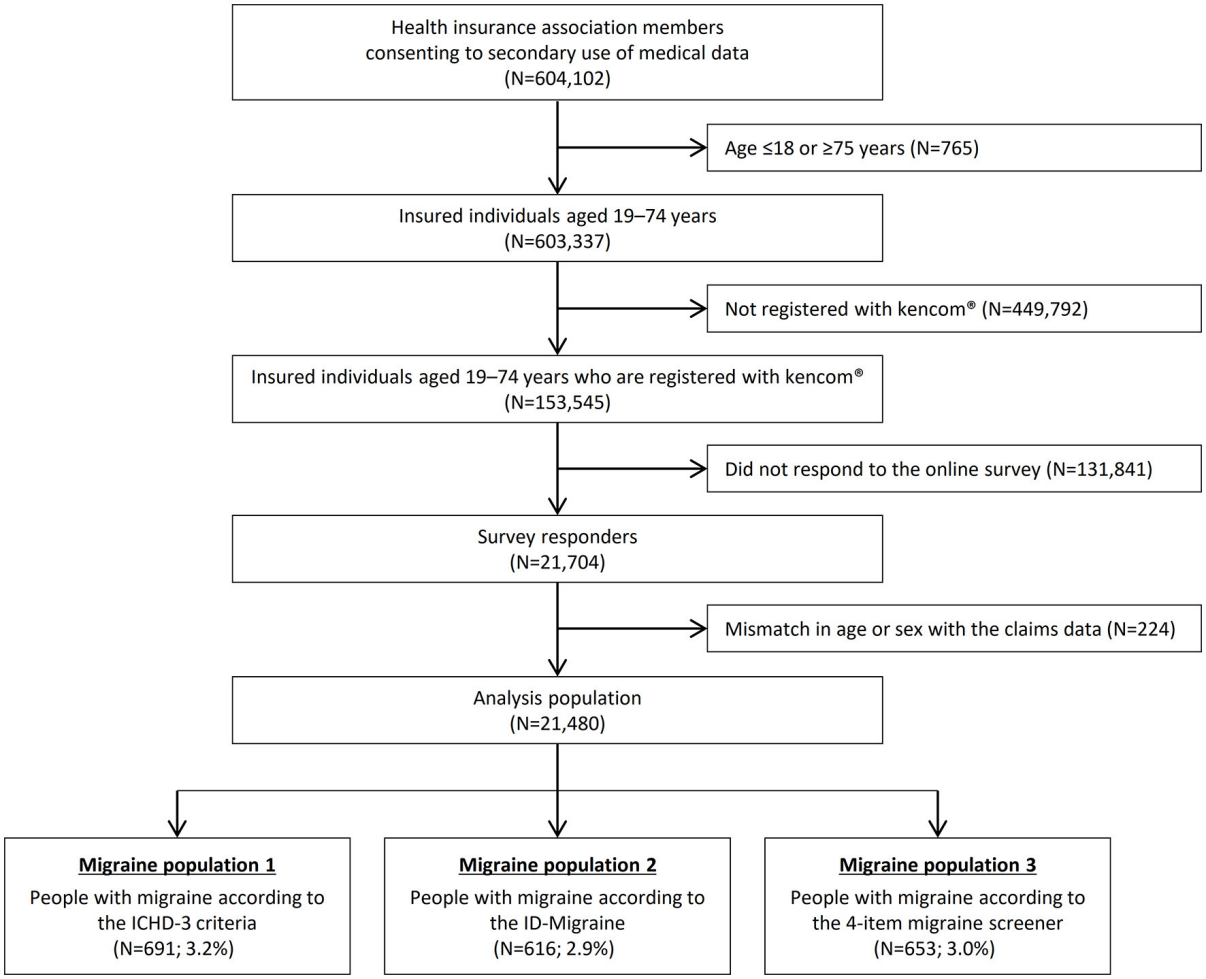
Disposition of participants included in the analyses.

A total of 604,102 members of the health insurance association consented to the secondary use of their medical data (Figure 1). After excluding 765 members aged <19 or >74 years, 603,337 (99.9%) aged 19–74 years met the age criteria. Of these, 153,545 (25.4%) were registered with Kencom^®^. Of the 21,704 individuals who responded to the survey, 224 were excluded because their age and/or sex did not match the medical claims data, resulting in an analysis population of 21,480 (99.0%) respondents (Figure 1).

### 3.2 Respondent demographic and disease characteristics

Of the 21,480 individuals included in the study population, 15,802 (73.6%) were men and 5,678 (26.4%) were women, with a mean (standard deviation) age of 48.8 (10.5) years (Table 3). Among the overall study population, 7,311 (34.0%) reported having headaches; of these, 735 (10.1%) responded that they had visited a physician for headaches, and 2,859 (39.1%) reported that they only used over-the-counter (OTC) drugs for headaches.

**Table 3:** Demographics and disease characteristics of the study population irrespective of the presence of headache.

	**Total (*n =* 21,480)**
**Age, years**	
Mean ± SD	48.8 ± 10.5
19–29 years	1,151 (5.4)
30–39 years	2,944 (13.7)
40–49 years	6,095 (28.4)
50–59 years	8,265 (38.5)
≥60 years	3,025 (14.1)
**Sex**	
Men	15,802 (73.6)
Women	5,678 (26.4)
**Headache**	**7,311 (34.0)**
**Number of headache days in the past 30 days** ^a^	
Mean ± SD	3.6 ± 4.7
0–3 days	5,387 (73.7)
4–7 days	1,082 (14.8)
8–14 days	488 (6.7)
≥15 days	354 (4.8)
Physician visit^a^	735 (10.1)
Use only OTC drugs for headaches^a^	2,859 (39.1)
**Migraine based on survey responses (ICHD-3)** ^b^	
Total	691 (100.0)
Episodic migraine	672 (97.3)
Chronic migraine	19 (2.7)
**Diagnosis records** ^c^	
Migraine	250 (1.2)
Tension-type headache	89 (0.4)
Premenstrual migraine	0 (0.0)
**Comorbidity** ^c^	
Hypertension	3,461 (16.1)
Cardiovascular disorders	1,321 (6.1)
Cerebrovascular disorders	534 (2.5)
Epilepsy	149 (0.7)
Gastrointestinal disorders	6,099 (28.4)
Constipation	1,055 (4.9)
Mood disorders	906 (4.2)
Anxiety disorders	479 (2.2)
Asthma	1,046 (4.9)
**Prescriptions** ^c^	
Acute medications (triptans, acetaminophen, and NSAIDs)	5,609 (26.1)
Prophylactic medications (valproic acid, topiramate, propranolol, lomerizine, and candesartan)	1,598 (7.4)

Data are n (%) unless otherwise stipulated.

a Proportions were calculated using 7,311 people with headache as the denominator.

b Proportions were calculated using 691 people with migraine according to the ICHD-3 criteria as the denominator.

c Data were obtained from medical claims. ICHD-3, International Classification of Headache Disorders, version 3; NSAIDs, non-steroidal anti-inflammatory drugs; OTC, over the counter; SD, standard deviation.

### 3.3 Assessment of claims-based algorithms

Of the 21,480 respondents, 691 (3.2%) had migraine according to the ICHD-3 criteria, 616 (2.9%) according to the ID-Migraine, and 653 (3.0%) according to the 4-item migraine screener (Figure 1). Using these results as the “true” diagnosis, we evaluated the ability of each algorithm to identify people with migraine.

#### 3.3.1 ICHD-3 criteria

The assessment indices calculated for each of the 12 algorithms using the ICHD-3 criteria for case ascertainment are summarized in Table 4. PPVs ranged from 19.2% for Algorithm 2 (at least one diagnostic record of migraine or tension-type headache) to 32.5% for Algorithm 9 [migraine diagnostic records more than once AND at least one prescription record for migraine prophylaxis or triptans in the same month as the diagnostic record of migraine (Algorithm 5)]. The overall sensitivity was low, with the highest value of 8.8% for Algorithm 2. In contrast, the specificity was excellent (~99%) for all algorithms, with high NPVs of ~97%.

**Table 4:** PPV, sensitivity, specificity, and NPV for each algorithm, using diagnosis according to the ICHD-3 criteria as true.

**Results based on the algorithm**		**Results according to the ICHD-3 criteria**		**PPV (%)**	**Sensitivity (%)**	**Specificity (%)**	**NPV (%)**
		**Yes**	**No**				
Algorithm 1	Yes	57	193	22.8	8.2	99.1	97.0
	No	634	20,596				
Algorithm 2	Yes	61	256	19.2	8.8	98.8	97.0
	No	630	20,533				
Algorithm 3	Yes	45	134	25.1	6.5	99.4	97.0
	No	646	20,655				
Algorithm 4	Yes	47	166	22.1	6.8	99.2	97.0
	No	644	20,623				
Algorithm 5	Yes	44	102	30.1	6.4	99.5	97.0
	No	647	20,687				
Algorithm 6	Yes	51	154	24.9	7.4	99.3	97.0
	No	640	20,635				
Algorithm 7	Yes	44	108	28.9	6.4	99.5	97.0
	No	647	20,681				
Algorithm 8	Yes	52	188	21.7	7.5	99.1	97.0
	No	639	20,601				
Algorithm 9	Yes	37	77	32.5	5.4	99.6	96.9
	No	654	20,712				
Algorithm 10	Yes	43	111	27.9	6.2	99.5	97.0
	No	648	20,678				
Algorithm 11	Yes	37	81	31.4	5.4	99.6	96.9
	No	654	20,708				
Algorithm 12	Yes	44	132	25.0	6.4	99.4	97.0
	No	647	20,657				

ICHD-3, International Classification of Headache Disorders, version 3; NPV, negative predictive value; PPV, positive predictive value.

#### 3.3.2 ID-Migraine

The evaluation results of each algorithm using the ID-Migraine for case ascertainment are summarized in Table 5. PPVs ranged from 26.2% for Algorithm 2 to 39.7% for Algorithm 5. The sensitivity ranged from 7.3% for Algorithms 9 and 11 (the same diagnostic records of migraine or tension-type headache more than once AND at least one prescription record for migraine prophylaxis or triptans in the same month as the diagnostic record of migraine or tension-type headache) to 13.5% for Algorithm 2. The specificity was high at ~99% for all algorithms, with high NPVs of ~97%.

**Table 5:** PPV, sensitivity, specificity, and NPV for each algorithm, using diagnosis according to the ID-Migraine as true.

**Results based on the algorithm**		**Results according to the ID-Migraine**		**PPV (%)**	**Sensitivity (%)**	**Specificity (%)**	**NPV (%)**
		**Yes**	**No**				
Algorithm 1	Yes	78	172	31.2	12.7	99.2	97.5
	No	538	20,692				
Algorithm 2	Yes	83	234	26.2	13.5	98.9	97.5
	No	533	20,630				
Algorithm 3	Yes	55	124	30.7	8.9	99.4	97.4
	No	561	20,740				
Algorithm 4	Yes	57	156	26.8	9.3	99.3	97.4
	No	559	20,708				
Algorithm 5	Yes	58	88	39.7	9.4	99.6	97.4
	No	558	20,776				
Algorithm 6	Yes	70	135	34.1	11.4	99.4	97.4
	No	546	20,729				
Algorithm 7	Yes	58	94	38.2	9.4	99.5	97.4
	No	558	20,770				
Algorithm 8	Yes	72	168	30.0	11.7	99.2	97.4
	No	544	20,696				
Algorithm 9	Yes	45	69	39.5	7.3	99.7	97.3
	No	571	20,795				
Algorithm 10	Yes	54	100	35.1	8.8	99.5	97.4
	No	562	20,764				
Algorithm 11	Yes	45	73	38.1	7.3	99.7	97.3
	No	571	20,791				
Algorithm 12	Yes	55	121	31.3	8.9	99.4	97.4
	No	561	20,743				

NPV, negative predictive value; PPV, positive predictive value.

#### 3.3.3 4-item migraine screener

The results of using the 4-item migraine screener for case ascertainment are summarized in Table 6. PPVs ranged from 27.1% for Algorithm 2 to 42.1% for Algorithm 9. The sensitivity was the lowest at 7.4% for Algorithms 9 and 11, whereas the highest was 13.2% for Algorithm 2. The specificity was ~99%, and the NPVs were ~97% for all algorithms.

**Table 6:** PPV, sensitivity, specificity, and NPV for each algorithm, using diagnosis according to the 4-item migraine screener as true.

**Results based on the algorithm**		**Results according to the 4-item migraine screener**		**PPV (%)**	**Sensitivity (%)**	**Specificity (%)**	**NPV (%)**
		**Yes**	**No**				
Algorithm 1	Yes	81	169	32.4	12.4	99.2	97.3
	No	572	20,658				
Algorithm 2	Yes	86	231	27.1	13.2	98.9	97.3
	No	567	20,596				
Algorithm 3	Yes	58	121	32.4	8.9	99.4	97.2
	No	595	20,706				
Algorithm 4	Yes	60	153	28.2	9.2	99.3	97.2
	No	593	20,674				
Algorithm 5	Yes	61	85	41.8	9.3	99.6	97.2
	No	592	20,742				
Algorithm 6	Yes	73	132	35.6	11.2	99.4	97.3
	No	580	20,695				
Algorithm 7	Yes	61	91	40.1	9.3	99.6	97.2
	No	592	20,736				
Algorithm 8	Yes	75	165	31.3	11.5	99.2	97.3
	No	578	20,662				
Algorithm 9	Yes	48	66	42.1	7.4	99.7	97.2
	No	605	20,761				
Algorithm 10	Yes	57	97	37.0	8.7	99.5	97.2
	No	596	20,730				
Algorithm 11	Yes	48	70	40.7	7.4	99.7	97.2
	No	605	20,757				
Algorithm 12	Yes	58	118	33.0	8.9	99.4	97.2
	No	595	20,709				

NPV, negative predictive value; PPV, positive predictive value.

Additional analyses based on cases that meet all three criteria of ICHD-3, ID-Migraine, and 4-item screener and those that meet one of them are provided in Supplementary Tables S2, S3. Both results showed that Algorithm 9 yielded the highest PPV, and Algorithm 2 yielded the highest sensitivity. This pattern remained consistent with that found among the migraine cases identified using each of the three methods.

#### 3.3.4 Stratification by sex and age groups

As Algorithm 9 yielded the highest (or the second highest) PPV regardless of the criteria used to judge “true” migraine cases, we additionally evaluated its performance, stratifying by sex and age groups (Tables 7–9). According to the ICHD-3 criteria, the PPVs were 17.3% for men and 45.2% for women when stratified by sex (Table 7). PPVs were the highest at 66.7% for people aged 19–29 years, followed by 42.1% for 30–39 years and 41.3% for 40–49 years, and they decreased to18.4% for those aged 50–59 years and 12.5% for those aged ≥60 years. Similar trends were observed with the ID-Migraine (Table 8) and the 4-item migraine screener (Table 9).

**Table 7:** Assessment results of algorithm 9, using diagnosis according to the ICHD-3 criteria as true.

**Category**	**Algorithm 9**	**ICHD-3**		**PPV (%)**	**Sensitivity (%)**	**Specificity (%)**	**NPV (%)**
		**Yes**	**No**				
Men (*N =* 15,802)	Yes	9	43	17.3	3.3	99.7	98.3
	Total	272	15,530				
Women (*N =* 5,678)	Yes	28	34	45.2	6.7	99.4	93.0
	Total	419	5,259				
19–29 years (*N =* 1,151)	Yes	2	1	66.7	3.3	99.9	94.9
	Total	60	1,091				
30–39 years (*N =* 2,944)	Yes	8	11	42.1	4.3	99.6	94.0
	Total	184	2,760				
40–49 years (*N =* 6,095)	Yes	19	27	41.3	7.3	99.5	96.0
	Total	262	5,833				
50–59 years (*N =* 8,265)	Yes	7	31	18.4	4.0	99.6	98.0
	Total	175	8,090				
≥60 years (*N =* 3,025)	Yes	1	7	12.5	10.0	99.8	99.7
	Total	10	3,015				

ICHD-3, International Classification of Headache Disorders, version 3; NPV, negative predictive value; PPV, positive predictive value.

**Table 8:** Assessment results of algorithm 9, using diagnosis according to the ID-Migraine as true.

**Category**	**Algorithm 9**	**ID-Migraine**		**PPV (%)**	**Sensitivity (%)**	**Specificity (%)**	**NPV (%)**
		**Yes**	**No**				
Men (*N =* 15,802)	Yes	8	44	15.4	3.8	99.7	98.7
	Total	208	15,594				
Women (*N =* 5,678)	Yes	37	25	59.7	10.0	99.5	94.0
	Total	371	5,245				
19–29 years (*N =* 1,151)	Yes	3	0	100.0	7.3	100.0	96.7
	Total	41	1,110				
30–39 years (*N =* 2,944)	Yes	9	10	47.4	5.5	99.6	94.7
	Total	164	2,780				
40–49 years (*N =* 6,095)	Yes	18	28	39.1	8.5	99.5	96.8
	Total	212	5,883				
50–59 years (*N =* 8,265)	Yes	13	25	34.2	7.0	99.7	97.9
	Total	187	8,078				
≥60 years (*N =* 3,025)	Yes	2	6	25.0	16.7	99.8	99.7
	Total	12	3,013				

NPV, negative predictive value; PPV, positive predictive value.

**Table 9:** Assessment results of algorithm 9, using diagnosis according to the 4-item migraine screener as true.

**Category**	**Algorithm 9**	**4-item migraine screener**		**PPV (%)**	**Sensitivity (%)**	**Specificity (%)**	**NPV (%)**
		**Yes**	**No**				
Men (*N =* 15,802)	Yes	8	44	15.4	3.7	99.7	98.7
	Total	216	15,586				
Women (*N =* 5,678)	Yes	40	22	64.5	9.2	99.6	92.9
	Total	437	5,241				
19–29 years (*N =* 1,151)	Yes	3	0	100.0	6.7	100.0	96.3
	Total	45	1,106				
30–39 years (*N =* 2,944)	Yes	9	10	47.4	5.2	99.6	94.4
	Total	172	2,772				
40–49 years (*N =* 6,095)	Yes	21	25	45.7	9.3	99.6	96.6
	Total	227	5,868				
50–59 years (*N =* 8,265)	Yes	13	25	34.2	6.6	99.7	97.8
	Total	196	8,069				
≥60 years (*N =* 3,025)	Yes	2	6	25.0	15.4	99.8	99.6
	Total	13	3,012				

NPV, negative predictive value; PPV, positive predictive value.

## 4 Discussion

In this study, we explored the ability of 12 coding algorithms, based on diagnostic records alone or in combination with prescription records, to identify people with migraine within a large-scale Japanese claims database. As migraine is diagnosed based primarily on patients' descriptions of their symptoms, we judged the “true” migraine cases using self-reported information collected in the online survey. The non-use of semi-structured interviews limited the accuracy of “true” migraine cases in this study. However, we observed consistent trends in the assessment of our algorithms for the three criteria used for case ascertainment. These trends, which are discussed below, can provide useful information for considering an appropriate coding algorithm to define migraines in each claims database study.

The 12 algorithms had overall low PPVs of ~20–30% with the ICHD-3 criteria and 30–40% with the ID-Migraine or 4-item migraine screener. However, this result should be interpreted in light of the fact that PPV depends on disease prevalence (26). These values were influenced by the low prevalence of migraine in this population (2.9–3.2%), which was lower than the reported prevalence of 6.0–8.6% in Japan (3, 4). This low prevalence may be related to the fact that this study was based on a self-administered survey without semi-structured interviews, which may have underestimated the prevalence of migraine and PPV. Many people with migraine are reportedly undiagnosed (3, 5, 27). A previous study reported that 59.4–71.8% of people with migraine in Japan had never consulted a physician previously, and only 11.6% were aware of the condition (28). Low awareness of migraine and the common use of OTC drugs for headaches (3) may have underestimated the number of people with migraine in the claims database. Indeed, only 250 people had a migraine diagnostic record in this study, whereas the responses to the survey suggested that 616–691 people possibly had migraines. This may also be partially responsible for the overall low sensitivity of our algorithms (5.4–13.5%), given the possibility of misclassifying patients who had migraines as “false negatives” because there are no migraine records in their claim records.

Among the 12 algorithms, we found that Algorithms 9 and 5 yielded the highest PPVs (32.5–42.1%), and this trend was consistent regardless of the criteria used for case ascertainment. This was probably because the strict conditions that require single (Algorithm 5) or multiple (Algorithm 9) diagnostic records plus prescription records of migraine prophylaxis or triptans could reduce “false positives.” Interestingly, the addition of acetaminophen or NSAIDs to triptans lowered the PPVs (Algorithms 6 vs. 5, 8 vs. 7, 10 vs. 9, and 12 vs. 11). These analgesics have wider indications and can be used for other conditions, which may have increased “false positives.” In contrast, Algorithm 2, which relied on a single diagnostic record of migraine or tension-type headache had the lowest PPV (19.2–27.1%). The algorithm used in a study that aims to estimate the relative risk of an outcome should have a high PPV so that the relative risk can be correctly estimated (26). Therefore, a strict coding algorithm based on a combination of multiple diagnostic records and prescription records of specific treatments, such as Algorithm 9, may be the most suitable for a migraine study with such an aim because it can reduce “false positives” and increase PPV.

However, a high PPV has high specificity, possibly at the sacrifice of sensitivity, as a result of increasing “false negatives (26).” Accordingly, Algorithm 9, which had high PPVs, had the lowest sensitivity (5.4–7.4%), while Algorithm 2, which had low PPVs, had the highest sensitivity (8.8–13.5%). For an exploratory study or a study that aims to estimate the prevalence or incidence of an outcome, an algorithm with high sensitivity is desirable (26) because maximizing the inclusion of patients with the target disease is more important than purifying the population under strict conditions. Therefore, for such a study aim, an algorithm based solely on a simple diagnostic record of migraine or tension-type headaches, such as Algorithm 2, may be desirable. This algorithm had the highest sensitivity, although at low levels, yet had an excellent specificity of ~99%, indicating the high accuracy of the diagnostic records in the claims database.

In stratified analyses, the PPV was higher in women and individuals aged 20 to 40 years, which is in line with the sex and age trends of migraine prevalence. The prevalence of migraine in women is 2 to 3.6 times higher than that in men (3, 29). Moreover, the prevalence is the highest between 30 and 39 years of age and decreases as age increases (30). Considering that the present study population was predominantly male (73.6%), the prevalence of migraine may have been lower than that in the general population, which may have affected the PPVs observed in our analysis of the overall study population. Therefore, our data stratified by sex and age group may be more useful as a reference when considering an appropriate claims-based algorithm in other studies, depending on the demographic characteristics of the population in these settings.

This study has some limitations, including those previously reported (23). For example, the study population may not represent the overall adult population of Japan since the database comprised data from employees and family members of large companies that are members of the health insurance associations, and survey respondents were limited to the Kencom^®^ users, with a high proportion of men (73.6%) compared to the general population in Japan [the prevalence of migraine in women and men was however 7.4 and 1.7%, respectively (23)]. These background characteristics may have influenced the low number of PPVs identified in this study. Because the prevalence of migraine or the distribution of other factors in our data may differ from those of other settings, the absolute values of this study may not be applicable to other databases.

One major limitation was that the present study used self-reported information collected in the online survey to judge the “true” migraine cases. Although the survey questionnaire included all items necessary to classify migraine according to the ICHD-3 criteria, no consultations with healthcare providers restricted us from obtaining an accurate or more reliable “true” status of migraine. Moreover, the ID-Migraine and 4-item migraine screener are screening tools although their use is recommended to aid diagnosis in clinical practice (25). However, these screening tools were used for supplemental purposes, considering that the ICHD-3 criteria may be too strict and miss unknown “true” migraineurs. Therefore, the accuracy of the case ascertainment in this study was limited, and the absolute values obtained should not be overinterpreted. Nevertheless, it is noteworthy that consistent trends were observed in the performance of the 12 algorithms across the three criteria. These findings will help us understand the advantages and disadvantages of each claims-based algorithm for use in migraine studies using Japanese claims databases.

## 5 Conclusion

A claims-based algorithm based on both the diagnostic records and prescription records of specific migraine medications had the highest PPV among the 12 algorithms considered, suggesting that such strict conditions may be appropriate for a study aimed at estimating relative risks. However, for an exploratory study, an algorithm based on a single diagnostic record of migraine or tension-type headache may be more suitable because it has higher sensitivity while maintaining high specificity. Because a suitable algorithm differs depending on the purpose of the study, it is important to choose an appropriate algorithm to define migraine in each claims database study.

## Data availability statement

The data that support the findings of this study are available from DeSC Healthcare, Inc. (Tokyo, Japan), but restrictions apply to the availability of these data, which were used under license for the current study and are not publicly available. However, data are available from the authors upon reasonable request and with permission of DeSC Healthcare, Inc. Requests to access these datasets should be directed to KY, Yamato.Kentaro@otsuka.jp.

## Ethics statement

The studies involving humans were approved by the Independent Ethics Committee of Otsuka Pharmaceutical Co., Ltd. (approval no. 220617). The study used anonymized data, and no new individual-level consent was obtained for data use. The survey was conducted in accordance with the Ethical Guidelines for Medical and Biological Research Involving Human Subjects in Japan and the Declaration of Helsinki (revised in October 2013) of the World Medical Association. The studies were conducted in accordance with the local legislation and institutional requirements. Written informed consent for participation was not required from the participants or the participants' legal guardians/next of kin in accordance with the national legislation and institutional requirements.

## Author contributions

KY and HS contributed to the study conception, design, and contributed to the manuscript drafting. KH and TN reviewed and revised it critically for important intellectual content. All authors approved the final version of the manuscript for publication and agreed to be accountable for all aspects of this study and made substantial contributions to the analysis or interpretation of data.
