# Supplementary material for: Validation and comparison of the coding algorithms to identify people with migraine using Japanese claims data
Source: Front Neurol. 2023 Nov 27;14:1231351. doi: 10.3389/fneur.2023.1231351 (PMC10711110; doi:10.3389/fneur.2023.1231351)
Supplement: Supplementary file 1 [file Table_1.DOCX]

**Supplementary Table S1. Conditions to be classified as migraine according to each of the three criteria (ICHD-3 criteria, ID-Migraine, and 4-item migraine screener)**

| Items | **ICHD-3** | | | **ID-Migraine** | | **4-item migraine screener** | | |
| --- | --- | --- | --- | --- | --- | --- | --- | --- |
| Duration (single answer) | *Condition 1* At least b), c), or d) is selected | | |  | |  | | |
| a) <4 hours |  | | |  | |  | | |
| b) Half a day | 〇 | | |  |  |  |  |  |
| c) All day |  |  |  |  |  |  |  |  |
| d) 2 to 3 days |  |  |  |  |  |  |  |  |
| e) 4 to 14 days |  | | |  |  |  |  |  |
| f) ≥15 days |  | | |  |  |  |  |  |
| Site of pain (multiple answers) | *Condition 2* At least a) is selected | | |  | |  | | |
| a) Unilateral | 〇 | | |  | |  | | |
| b) Bilateral |  | | |  |  |  |  |  |
| c) Frontal |  | | |  |  |  |  |  |
| d) Occipital |  | | |  |  |  |  |  |
| e) Periorbital |  | | |  |  |  |  |  |
| f) Other |  | | |  |  |  |  |  |
| Characteristics (multiple answers) | *Condition 3*  At least a) or g) is selected | | |  | |  | | |
| a) Throbbing or pulsating pain | 〇 | | |  | |  | | |
| b) Tightening pain |  | | |  |  |  |  |  |
| c) Prickling pain |  | | |  |  |  |  |  |
| d) Tingling pain |  | | |  |  |  |  |  |
| e) Gouged pain behind the eye |  | | |  |  |  |  |  |
| f) Burning pain |  | | |  |  |  |  |  |
| g) Pounding pain^2^ | 〇 | | |  |  |  |  |  |
| h) Cracking pain (like being hit by a hammer) |  | | |  |  |  |  |  |
| i) Heavy-headed |  | | |  |  |  |  |  |
| j) Other |  | | |  |  |  |  |  |
| Change in severity due to daily activities (walking, climbing up-stairs, etc.) or due to physical activity (single answer) | *Condition 4*  At least a) in “Change in severity due to daily activities” or a) in “State when in pain” is selected | | | *Condition 1* At least a) in “Change in severity due to daily activities” or a) in “State when in pain” is selected | | *Condition 1*  At least a) in “Change in severity due to daily activities” or a) in “State when in pain” is selected | | |
| a) Worsens (avoid movement due to pain) | 〇 | | | 〇 | | 〇 | | |
| b) No change |  | | |  | |  | | |
| c) Gets better |  | | |  | |  | | |
| d) Sometimes gets better and sometimes gets worse |  | | |  | |  | | |
| e) I don't know |  | | |  | |  | | |
| State when in pain (single answer) | *Condition 4*  At least a) in “Change in severity due to daily activities” or a) in “State when in pain” is selected | | | *Condition 1*  At least a) in “Change in severity due to daily activities” or a) in “State when in pain” is selected | | *Condition 1*  At least a) in “Change in severity due to daily activities” or a) in “State when in pain” is selected | | |
| a) It is more comfortable to stay still | 〇 | | | 〇 | | 〇 | | |
| b) Staying still does not change the severity of pain |  | | |  | |  | | |
| c) Pain makes it hard to stay still |  | | |  | |  | | |
| d) I don't know |  | | |  | |  | | |
| Symptom associated with headache (multiple answers) | *Condition 5*  At least include any of the following combinations | | | *Conditions* | | *Conditions* | | |
|  |  |  |  | *2*  At least a) is selected | *3*  At least b) is selected | *2*  At least a) is selected | *3*  At least b) is selected | *4*  At least d) is selected |
|  | a) | b) and c) | a), b), and c) |  |  |  |  |  |
| a) Nausea or vomiting | 〇 |  | 〇 | 〇 |  | 〇 |  |  |
| b) Photophobia |  | 〇 | 〇 |  | 〇 |  | 〇 |  |
| c) Phonophobia |  | 〇 | 〇 |  |  |  |  |  |
| d) Osmophobia |  |  |  |  |  |  |  | 〇 |
| e) Bloodshot eye on the side of headache |  |  |  |  |  |  |  |  |
| f) Teary eye on the side of headache |  |  |  |  |  |  |  |  |
| g) Runny nose on the side of headache |  |  |  |  |  |  |  |  |
| h) Dizziness |  |  |  |  |  |  |  |  |
| i) Weakness or lethargy |  |  |  |  |  |  |  |  |
| j) Stiff shoulders |  |  |  |  |  |  |  |  |
| k) Stiff neck |  |  |  |  |  |  |  |  |
| l) Numbness in hands and feet |  |  |  |  |  |  |  |  |
| m) Other |  |  |  |  |  |  |  |  |
| Severity (when not taking medicines) (single answer) | *Condition 6*  At least one of c), d), or e) is selected | | |  | |  | | |
| a) No pain |  | | |  | |  | | |
| b) Little pain |  | | |  |  |  |  |  |
| c) Moderate pain | 〇 | | |  |  |  |  |  |
| d) Quite a bit of pain |  |  |  |  |  |  |  |  |
| e) Extreme pain |  |  |  |  |  |  |  |  |
| **Diagnosis of migraine according to each diagnostic criteria/screening tools** | The patient meeting the 5 (probably migraine) or all 6 conditions was considered to have migraine. | | | The patient meeting at least 2 of the 3 conditions was considered to have migraine. | | The patient meeting at least 2 of the 4 conditions was considered to have migraine. | | |
