# Supplementary material for: Validation and comparison of the coding algorithms to identify people with migraine using Japanese claims data
Source: Front Neurol. 2023 Nov 27;14:1231351. doi: 10.3389/fneur.2023.1231351 (PMC10711110; doi:10.3389/fneur.2023.1231351)
Supplement: Supplementary file 2 [file Table_2.DOCX]

**Supplementary Table S2. PPV, sensitivity, specificity, and NPV for each algorithm, using diagnosis meeting all three methods of the ICHD-3 criteria, the ID-Migraine, and the 4-item migraine screener as true**

| **Results based on the algorithm** | | **Results according to the ICHD-3 criteria, the ID-Migraine, and the 4-item migraine screener** | | **PPV (%)** | **Sensitivity (%)** | **Specificity (%)** | **NPV (%)** |
| --- | --- | --- | --- | --- | --- | --- | --- |
|  |  | Yes | No |  |  |  |  |
| Algorithm 1 | Yes | 42 | 208 | 16.8 | 12.7 | 99.0 | 98.6 |
|  | No | 290 | 20,940 |  |  |  |  |
| Algorithm 2 | Yes | 45 | 272 | 14.2 | 13.6 | 98.7 | 98.6 |
|  | No | 287 | 20,876 |  |  |  |  |
| Algorithm 3 | Yes | 32 | 147 | 17.9 | 9.6 | 99.3 | 98.6 |
|  | No | 300 | 21,001 |  |  |  |  |
| Algorithm 4 | Yes | 33 | 180 | 15.5 | 9.9 | 99.1 | 98.6 |
|  | No | 299 | 20,968 |  |  |  |  |
| Algorithm 5 | Yes | 34 | 112 | 23.3 | 10.2 | 99.5 | 98.6 |
|  | No | 298 | 21,036 |  |  |  |  |
| Algorithm 6 | Yes | 37 | 168 | 18.0 | 11.1 | 99.2 | 98.6 |
|  | No | 295 | 20,980 |  |  |  |  |
| Algorithm 7 | Yes | 34 | 118 | 22.4 | 10.2 | 99.4 | 98.6 |
|  | No | 298 | 21,030 |  |  |  |  |
| Algorithm 8 | Yes | 37 | 203 | 15.4 | 11.1 | 99 | 98.6 |
|  | No | 295 | 20,945 |  |  |  |  |
| Algorithm 9 | Yes | 28 | 86 | 24.6 | 8.4 | 99.6 | 98.6 |
|  | No | 304 | 21,062 |  |  |  |  |
| Algorithm 10 | Yes | 31 | 123 | 20.1 | 9.3 | 99.4 | 98.6 |
|  | No | 301 | 21,025 |  |  |  |  |
| Algorithm 11 | Yes | 28 | 90 | 23.7 | 8.4 | 99.6 | 98.6 |
|  | No | 304 | 21,058 |  |  |  |  |
| Algorithm 12 | Yes | 31 | 145 | 17.6 | 9.3 | 99.3 | 98.6 |
|  | No | 301 | 21,003 |  |  |  |  |

Abbreviations: ICHD-3, International Classification of Headache Disorders, version 3; NPV, negative predictive value; PPV, positive predictive value
