# Supplementary material for: Validation and comparison of the coding algorithms to identify people with migraine using Japanese claims data
Source: Front Neurol. 2023 Nov 27;14:1231351. doi: 10.3389/fneur.2023.1231351 (PMC10711110; doi:10.3389/fneur.2023.1231351)
Supplement: Supplementary file 3 [file Table_3.DOCX]

**Supplementary Table S3. PPV, sensitivity, specificity, and NPV for each algorithm, using diagnosis meeting one of the methods of the ICHD-3 criteria, the ID-Migraine, or the 4-item migraine screener as true**

| **Results based on the algorithm** | | **Results according to the ICHD-3 criteria, the ID-Migraine, or the 4-item migraine screener** | | **PPV (%)** | **Sensitivity (%)** | **Specificity (%)** | **NPV (%)** |
| --- | --- | --- | --- | --- | --- | --- | --- |
|  |  | Yes | No |  |  |  |  |
| Algorithm 1 | Yes | 93 | 157 | 37.2 | 9.3 | 99.2 | 95.7 |
|  | No | 912 | 20318 |  |  |  |  |
| Algorithm 2 | Yes | 99 | 218 | 31.2 | 9.9 | 98.9 | 95.7 |
|  | No | 906 | 20257 |  |  |  |  |
| Algorithm 3 | Yes | 68 | 111 | 38.0 | 6.8 | 99.5 | 95.6 |
|  | No | 937 | 20364 |  |  |  |  |
| Algorithm 4 | Yes | 71 | 142 | 33.3 | 7.1 | 99.3 | 95.6 |
|  | No | 934 | 20333 |  |  |  |  |
| Algorithm 5 | Yes | 68 | 78 | 46.6 | 6.8 | 99.6 | 95.6 |
|  | No | 937 | 20397 |  |  |  |  |
| Algorithm 6 | Yes | 84 | 121 | 41.0 | 8.4 | 99.4 | 95.7 |
|  | No | 921 | 20354 |  |  |  |  |
| Algorithm 7 | Yes | 68 | 84 | 44.7 | 6.8 | 99.6 | 95.6 |
|  | No | 937 | 20391 |  |  |  |  |
| Algorithm 8 | Yes | 87 | 153 | 36.3 | 8.7 | 99.3 | 95.7 |
|  | No | 918 | 20322 |  |  |  |  |
| Algorithm 9 | Yes | 54 | 60 | 47.4 | 5.4 | 99.7 | 95.5 |
|  | No | 951 | 20415 |  |  |  |  |
| Algorithm 10 | Yes | 66 | 88 | 42.9 | 6.6 | 99.6 | 95.6 |
|  | No | 939 | 20387 |  |  |  |  |
| Algorithm 11 | Yes | 54 | 64 | 45.8 | 5.4 | 99.7 | 95.5 |
|  | No | 951 | 20411 |  |  |  |  |
| Algorithm 12 | Yes | 68 | 108 | 38.6 | 6.8 | 99.5 | 95.6 |
|  | No | 937 | 20367 |  |  |  |  |

Abbreviations: ICHD-3, International Classification of Headache Disorders, version 3; NPV, negative predictive value; PPV, positive predictive value
